# Call for papers for a special issue on advanced diagnostics to inform public health policy

**DOI:** 10.2807/1560-7917.ES.2017.22.41.171012-1

**Published:** 2017-10-12

**Authors:** Eurosurveillance editorial team

**Affiliations:** 1 European Centre for Disease Prevention and Control (ECDC), Stockholm, Sweden

*Eurosurveillance* invites authors to submit papers in the categories, Research articles, Surveillance and Outbreak reports, Reviews and Perspectives for a special issue on advanced diagnostics to inform public health policy.

Recent advances in laboratory technology, including the application of qualitative and quantitative molecular diagnostics, proteomics (e.g. MALDI-TOF MS) and genomics, particularly whole genome microbial sequencing (WGS) and metagenomics are transforming the field of clinical and public health microbiology. The use of advanced methods enables unprecedented capability and capacity for rapid and comprehensive pathogen detection, quantitation and characterisation, when applied to clinical and environmental samples. Advanced diagnostics may therefore impact public health policy during specific incidents or on a wider scale.

Topics of interest may include, but are not limited to:

• Focused reviews portraying pathogen- or disease-specific use of advanced diagnostics in public health microbiology or practical aspects of integrating new diagnostics in public health;
• Advanced laboratory tools for generating data that inform risk assessment and risk management;
• Possible role of rapid diagnostic testing in public health and cross-border health security;
• Moving to culture-independent public health microbiology;
• Policy and Regulatory aspects of harnessing new technologies for public health.

The submission deadline is 15 April 2018. If you would like to submit a paper or ask for more information, please see our instructions for authors regarding article formats or contact the editorial team at eurosurveillance@ecdc.europa.eu.

